# Global prevalence of *Tritrichomonas foetus* across different animal species: A protocol for a systematic review and meta-analysis

**DOI:** 10.1371/journal.pone.0355688

**Published:** 2026-08-25

**Authors:** Madalitso Chelenga, Hosea Katete, Muonaouza Deleza, Lawrence Banda, John Kothowa, Muloongo C. Sitali

**Affiliations:** 1 Department of Veterinary Biomedical Sciences, Lilongwe University of Agriculture and Natural Resources, Lilongwe, Malawi; 2 Department of Veterinary Public Health and Epidemiology, Faculty of Veterinary Medicine, Lilongwe University of Agriculture and Natural Resources, Lilongwe, Malawi; 3 Department of Veterinary Clinical Studies, Faculty of Veterinary Medicine, Lilongwe University of Agriculture and Natural Resources, Lilongwe, Malawi; 4 Department of Biomedical Sciences, School of Veterinary Medicine, The University of Zambia, Lusaka, Zambia; University of Jeddah, SAUDI ARABIA

## Abstract

**Background:**

*Tritrichomonas foetus* is a protozoan parasite of global importance that causes significant reproductive disease in cattle and chronic diarrhea in cats, while commonly existing as a commensal in pigs. Emerging evidence of genetic diversity, transmission, and drug resistance necessitates a comprehensive understanding of its global prevalence. Here, we outline the protocol for a systematic review and meta-analysis to estimate the global prevalence of *T. foetus* in cattle, cats, and pigs.

**Methods:**

A comprehensive search of electronic databases, including PubMed, Scopus, Web of Science, CAB abstracts, and Embase, using a predefined search strategy, will be conducted to capture relevant studies. Observational studies reporting the prevalence or incidence of *T. foetus* infection in cattle, cats, or pigs will be included in the meta-analysis. There will be no language or geographic restrictions. Study selection, data extraction, and risk of bias assessment will be performed independently by at least two reviewers. The random-effects meta-analyses will be conducted to pool prevalence estimates, stratified by animal species and geographical regions. Subgroup analysis based on diagnostic methods and other animal- and management-related covariates will be conducted. Heterogeneity will be explored through subgroup analyses. Sensitivity analysis will be conducted using the leave-one-out technique.

**Discussion:**

We describe a protocol for a global prevalence meta-analysis of *T. foetus*, providing crucial insights into its epidemiology across different host species and regions. The findings are essential to inform the status of surveillance efforts and highlight knowledge gaps, necessary for developing effective control strategies and guiding future research across the vulnerable domestic animal species.

**Systematic review registration:**

International Prospective Register of Systematic Reviews (PROSPERO) registration number CRD420261303496.

## Background

Since the first identification of *Tritrichomonas foetus* as a cause of bovine infertility and abortion by the Italian scientist, Mazzanti in 1900 [1], contemporary research has revealed the complex nature of this protozoan parasite, particularly its host adaptation, genetic identity, transmission, and drug resistance. Beyond colonizing the preputial cavity and urogenital tract of bulls and cows, respectively, it is now known that *T. foetus* also colonizes the intestinal tract of cats to cause chronic large bowel diarrhea [2–6], as well as the nasal cavity, stomach, and intestines of pigs, where it causes no noticeable infection [7–9]. *T. foetus* has also been detected in the feces of dogs with diarrhea in one report [10]. Early cross-infection [11] and genome sequencing studies [8,12,13] and, more recently, advanced omics [14] have generated conflicting evidence regarding the genetic identity, host adaptation, and drug resistance of *T. foetus* isolates [15]. Notably, while the sequencing of variable DNA regions and comparison of gene sequences revealed no differences between porcine and bovine isolates [8], other studies identified consistent genetic divergence between the bovine/porcine isolates and the feline genotype [12,13]. These genetic differences may translate into phenotypic traits related to infectivity and pathogenicity, as it has been shown that bovine and porcine isolates are poorly infectious in cats, and vice versa [11]. Similarly, Gookin et al [16] found no association between *T. foetus* infection in cats and proximity to cattle. While these findings entail a species-specific distinction between bovine/porcine and cat genotypes, studies using comparative transcriptomics and proteomics found no molecular-level divergence between the isolates, suggesting that the feline and bovine/porcine isolates may belong to the same species [12,14,17–20]. This complexity is further complicated by the recent discovery of a third distinct ‘Southern Africa’ genotype in a bull in Namibia, whose broader distribution and clinical significance remain unknown [21].

The transmission of *T. foetus* in cattle, cats, and pigs presents another complexity in the epidemiology of this parasite. While venereal transmission is well-known in cattle, fecal-oral transmission via the ingestion of trophozoites is the primary route in cats [16,22]. However, the finding that *T. foetus* has also been isolated from the uterus of a cat with pyometra [23] raises questions regarding the possibility of reproductive tract colonization in this species. Yet a study evaluating the reproductive organs of purebred cats located in high endemic areas found no evidence of colonization by *T. foetus* [24]. A previous study demonstrated the *T. foetus’s* ability to form resistant mononucleate pseudocysts or cyst-like structures under adverse conditions, enabling it to survive for several days in environments such as water or feces [25]. This environmental resilience suggests that the fecal-oral transmission in domestic animals is possible, potentially explaining field reports of *T. foetus* in samples obtained from virgin bulls or its presence in herds where negative bulls co-mingled with positive cows, even when natural mating is used [26]. Further, *T. foetus* can be mechanically transmitted to naive cows during artificial insemination (AI) or vaginal examination, suggesting that the iatrogenic route cannot be ruled out completely [27]. Therefore, the presence of *T. foetus* in livestock and companion animals entails that human exposure, particularly through fecal contamination, could also be possible. However, the true extent of this zoonotic risk remains undefined. To date, only four opportunistic cases where *T. foetus* was isolated from immunocompromised patients have been reported [28–31]. In one of these cases, the patient had contact with farm animals, suggesting a possible occupational risk [29].

Globally, the prevalence of *T. foetus* seems to be context-dependent. *T. foetus* is known to be prevalent in regions where natural service is common [32], but low incidence rates have been reported in regions with intensive AI programs [32]. While *T. foetus* is more prevalent in older bulls, this age predisposition is the complete opposite in cats, where younger cats, particularly those kept in groups, tend to be more susceptible, with older cats only acting as asymptomatic carriers [4]. The country-specific prevalence estimates may also be influenced by control programs implemented. In cattle, control programs are based on the testing and exclusion of infected bulls due to the lack of effective treatment options against *T. foetus* [33]. In some developed countries, *T. foetus* is rarely diagnosed at all, due to the predominance of intensive production systems, semen testing before AI, and culling of infected bulls [34]. The ability of *T. foetus* to establish persistent infections by modulating or evading host immune responses, coupled with the documented emergence of resistance to standard antiprotozoal therapies, presents a huge bottleneck in the control of this pathogen [35].

Due to the economic and veterinary importance of *T. foetus*, as well as the complexities in its epidemiology, a global understanding of the prevalence estimates is necessary. Here, we describe the protocol to support the development of a systematic review and meta-analysis aiming at estimating the global prevalence of *T. foetus* infection in cattle, cats, and pigs. Concurrently, the protocol outlines an approach for establishing the relationship between the prevalence estimates (including the trends of occurrence) and such factors as breed/species predisposition, geographical region, management practices, diagnostic methods, and other relevant population characteristics. By stratifying the prevalence estimates by these factors, the protocol sets a precedent for investigating the potential sources of heterogeneity in prevalence estimates across studies, thereby informing the contexts requiring further research or strengthening the surveillance essential to control the spread of this parasite.

## Methods

### Protocol registration and reporting

This protocol is for a prospective systematic review and meta-analysis, prepared following the guidelines specified in the Cochrane Handbook [36] and is reported in line with the Preferred Reporting Items for Systematic Reviews and Meta-Analyses extension for Protocols (PRISMA-P) guidelines [37]. The protocol has been registered in the International Prospective Register of Systematic Reviews (PROSPERO) with the registration number CRD420261303496. All procedures, from reference search to data analysis, will be completed within a month (30 days).

### Inclusion and exclusion criteria

The Population, Exposure, Comparator, and Outcomes (PECO) framework has been developed to guide the inclusion and exclusion criteria as described elsewhere [38]. The specific domains are as described below.

1. *Population:* Cattle (*Bos taurus* or *Bos indicus*), domestic cats (*Felis catus*), and pigs (*Sus scrofa domesticus*) of any breed, age, sex, management system (e.g., intensive, extensive, household pets) reared globally;
2. *Exposure:* Infection with *T. foetus*. Studies that do not report prevalence or any other data from which prevalence can be calculated will be excluded from this study;
3. *Comparison:* None required for prevalence meta-analysis;
4. *Outcomes:* The primary outcome is the prevalence of *T. foetus* infection, defined as the proportion of the population infected with *T. foetus* at a specific point in time (point prevalence) or during a period (period prevalence) [39]. Infection status leading to the determination of prevalence must be confirmed using one or more of the following diagnostic methods: 1) Direct microscopic examination (e.g., wet mount, stained smear), 2) Culture using specific media (e.g., InPouch™ TF, modified Diamond’s medium), 3) Molecular tests, e.g., the polymerase chain reaction (PCR) targeting specific genes (e.g., 5.8S rRNA gene, cysteine proteases); and
5. *Study design:* We will include observational studies that report the prevalence, incidence, or detection of *T. foetus* in cattle, pigs, or cats. Any other study designs, such as randomized controlled trials (RCTs) and cross-infection studies, will be excluded from this study.

### Search strategy

A comprehensive literature search will be conducted across five electronic databases, including PubMed, Scopus, Web of Science, CAB Abstracts, and Embase. The search strategy will combine search terms following the PECOs framework outlined above. Further, grey searches will be conducted to capture conference abstracts, government surveillance reports, including ProQuest Dissertations, OIE reports, and FAO documents. Only peer-reviewed original studies will be included. The reference lists of all included studies and relevant review articles will be hand-searched for additional eligible studies that the original search might have missed. Reviews will be excluded from the study. The following search string will be used, first on PubMed, then adapted to the other databases based on their requirements:

(“Tritrichomonas foetus” OR “Trichomonas foetus” OR “T. foetus” OR “Bovine trichomoniasis” OR “Feline trichomonosis” OR Trichomonad OR “Tritrichomonas suis”) AND (Cattle OR Bovine OR Cow OR Heifer OR Bull OR Feline OR Cat OR Queen OR Porcine OR Pig OR Swine OR Boar OR Sow) AND (Prevalence OR “prevalence rate” OR Incidence OR “incidence rate” OR Isolation OR Identification OR Detection OR Cases OR Epidemiolog* OR “risk factor” OR transmission OR Infection OR diagnosis)

### Study selection and data management

All records retrieved from studies will be exported to the Covidence systematic review software platform to resolve duplicates and screen for eligibility. Each study will have its title and abstract screened independently by two reviewers using the predefined inclusion and exclusion criteria given above. There will be no language or date restrictions in this study to document a comprehensive trend analysis of the prevalence estimates. Non-English studies will be translated into English using appropriate translation tools. To resolve any potential misclassification bias, studies will be excluded if translation quality cannot be assured. Any conflicting findings will be resolved by engaging a third reviewer.

### Data extraction

Two reviewers will independently extract data using a standardized, pre-tested extraction form developed in Microsoft Excel. The following information will be extracted from each included study:

1. Study characteristics including Author (s), year of publication, country, study design, setting (e.g., farm, shelter, veterinary clinic, community), sampling method, and sample size.
2. Population characteristics: Host species, breed, age, sex, management system (e.g., dairy, beef, indoor/outdoor cat, intensive/extensive pig farm), clinical status (e.g., diarrheic, reproductive problem, healthy).
3. Exposure: Infection with *T. foetus*
4. Diagnostic techniques: Specimen type (e.g., preputial scraping/fluid, feces, vaginal swab, intestinal content), diagnostic test (s) used (e.g., culture, PCR, microscopy), and any relevant test details (e.g., primer/probe sequences for PCR).
5. Outcome data: Prevalence data on *T. foetus* stratified by animal species and geographical locations as the main effect outcomes. Additional outcomes will include prevalence estimates based on diagnostic tests, management practices (e.g., insemination practices, animal densities, etc.), population characteristics, and study characteristics. For molecular studies, data on the genetic types or strains identified will be extracted.

If data is missing or unclear, the corresponding authors will be contacted once via email. In a case where we don’t get responses from authors, the study will be excluded from the meta-analysis.

### Assessment of risk of bias and certainty of evidence

The Joanna Briggs Institute (JBI) critical appraisal checklists for observational epidemiological studies reporting prevalence and cumulative incidence data [40,41] will be used to assess the risk of bias in this study. All included studies will be critically appraised independently by two investigators. While any unanimous decision by both investigators on the quality of the studies will be considered final, discrepancies will be resolved by consensus. Studies with an overall score higher than 70% will be classified as having a high quality, those with a score between 50 and 70% as having medium quality, and those with a score less than 50% as having low quality, as previously described elsewhere [42]. To assess the impact of the difference in the quality of the studies (i.e., high, medium, and low), sensitivity and subgroup analyses based on risk-of-bias scores will be conducted.

The certainty of the evidence will be assessed using the GRADE framework (Grading of Recommendations Assessment, Development, and Evaluation), which considers the study design, risk of bias, imprecision, inconsistency, indirectness, and publication bias to rate the level of evidence [43]. The evidence will be rated as high, moderate, low, or very low [43]. Two independent investigators will assess the certainty of the included studies.

### Data analysis and interpretation

All the statistical analyses will be performed using the meta package in R 4.5.3. Firstly, prevalence proportion data from individual studies will be transformed using the Freeman-Tukey double arcsine transformation to stabilize variances, and the continuity correction will be applied to handle zero prevalence cases [44,45]. The prevalence of *T. foetus* will then be pooled using a DerSimonian and Laird random-effects model based on the inverse variance methods for combining results across different studies [46] because we anticipate variability both between and within studies [47]. For studies that do not directly report prevalence, available data such as the number of cases and total sample size, as well as incidence rates with duration of the presence of the disease in the population at risk, will be used to calculate prevalence. We anticipate conducting several main analyses stratified firstly by host species (i.e., cattle, cats, and pigs) and geographical regions. Due to the disparities in transmission routes, diagnostic specimens, clinical presentations, and epidemiological contexts, the prevalence estimates will be presented per distinct host species and not as a single global prevalence. Further main analysis per host species will be stratified by Geographical region (based on the WHO, i.e., Africa, Asia, Europe, Latin America and the Caribbean, North America, Oceania) and countries. Sub-group analyses will be performed by stratifying the prevalence estimates per animal species, by age groups, breeds within each species, animal density, management practices (for cattle: use of AI vs. natural mating), year of publication, and diagnostic techniques. In cases where studies report results from multiple tests, the data will be included in the analysis based on the following hierarchy: PCR > culture > microscopy [48]. Pooled estimates will be back-transformed and presented as percentages with 95% confidence intervals and prediction intervals.

Heterogeneity across studies will be assessed using Cochran’s Q test and the I^2^ statistic [49]. A p-value < 0.05 in Cochran’s Q test will indicate significant heterogeneity, while I^2^ values ≥ 50% will be interpreted as substantial heterogeneity. Values < 50% will reflect low heterogeneity. The sources of heterogeneity will be assessed via both subgroup analyses and sensitivity analyses. The latter will also be conducted to assess the robustness of the prevalence estimates using the modified leave-one-out approach, incorporating the iterative outlier detection [50]. A two-tailed p < 0.05 will be defined as statistical significance. The publication bias will be assessed by analyzing the asymmetry of funnel plots and by applying the Egger regression test [51]. However, funnel plots and Egger’s test have limited power when there are fewer than 10 studies [52]. In this case, we will not interpret Egger’s test p-values or funnel plot asymmetry as definitive evidence for or against publication bias. To strengthen the translational impact of the results generated in this study, we will engage some practicing small and large-animal veterinarians within our network to draw insights and strengthen our interpretation of the results.

## Discussion

This protocol describes the detailed approach to a prospective systematic review and meta-analysis (SR) aiming at generating data describing the global prevalence of *T. foetus* across multiple domestic animal species around the globe. By pre-defining the study selection criterion, search strategy, assessment of risk of bias, and assessment of certainty of evidence, the protocol sets a strong precedent allowing for the reproducibility of the prospective SR. The review subsequently clarify the gray areas related to *T. foetus* epidemiology, particularly how the complex genetic diversity, transmission, environmental survival, immune evasion, and antiprotozoal drug resistance domains translate to the pooled prevalence estimates across different geographical regions, livestock characteristics (e.g., species, breeds, age, etc.) and management practices (e.g., breeding strategies, stocking densities, diagnostic capabilities and control measures). The region-specific prevalence estimates will further provide insights regarding regions with a high disease burden, thereby identifying areas where interventions could be better prioritized. Further, the country-specific prevalence estimates for cattle will be essential to justify why countries need to invest in the surveillance and control programs (such as bull testing and culling, or vaccination), which are often ignored in resource-limited settings. Similarly, a better understanding of the prevalence of feline tritrichomoniasis will be an essential stimulus towards strengthening diagnostic and management protocols aimed at controlling the spread of *T. foetus*-induced chronic diarrhoea to the populations at risk.

Despite its methodological vigor, the implementation of this protocol faces potential limitations that might affect the accurate interpretation of the global prevalence of *T. foetus*. Notably, some countries may not test or publish cases and prevalence rates of *T. foetus* in domestic animals due to a lack of robust surveillance systems. Should this be the case, the results will be reported cautiously, factoring in this phenomenon in the interpretation of the results. Further, the anticipated variations in study designs, settings, study populations, sampling strategies, diagnostic tests, and quality scores present a potential bottleneck that may complicate the direct comparisons and pooling of prevalence estimates. In particular, the sensitivity and specificity of different diagnostic tests (i.e., direct microscopy, culture, and PCR) vary enormously, in addition to the fact that different samples and tests are used across domestic animal species. To address this issue, the main analysis will be conducted by distinct animal species and geographical regions rather than pooling the prevalence values into a single global prevalence estimate. The subgroup and sensitivity analyses will also be essential to address the methodological and contextual sources of heterogeneity in the prospective SR.

## Supporting information

S1 Checklist PRISMA-P 2015 checklist. (PDF)
